# Iodized Phenol—“Battey’s Formula”—in Eczema Marginatum

**Published:** 1880-04

**Authors:** W. J. H. Bellamy

**Affiliations:** Wilmington, N. C.


### IODIZED PHENOL—"BATTEY'S FORMULA"—IN ECZEMA MARGINATUM.

BY W. J. H. BELLAMY, M. D., WILMINGTON, N. C.

℞

Iodinii cryst, ℥ ss.

Ac. Carbolic cryst, ℥ j.

Mix and combine the two by gentle heat.

The above formula of the renowned Battey of Rome, Ga., provides a combination which has probably given more satisfaction to the gynecologist in uterine therapeutics than any agent that has been suggested for many years past. It is not alone useful for such affections, as concerns the specialist alluded to, but as I am prepared by quite an extensive experience with its use to say it has given me more satisfaction in the management of those intractable forms of skin disease characterized by intolerable itching, than any of the much vaunted parasiticides so much in use by the dermatologists of the present day. Most particularly in that disease, the pathology of which is now well understood, viz: eczema marginatum, and which, for such a long time has almost baffled the skill of the country practitioner, is this agent most useful. In most cases of "skin disease," when the diagnosis is not clear, but where itching is the prominent symptom, and when it is reasonable to suppose the presence of some parasite, it is a most useful remedy. It allays itching, it relieves pain. The anæsthetic property of the carbolic acid prevents the agents from giving much more than momentary pain.

My rule has been at first to dilute it with glycerine, equal parts, making the application twice in twenty-four hours, touching every point of irritation thoroughly by means of an ordinary camel's hair pencil, or glass rod (brush.) It may be used according to the sensibility and idiosyncrasy of each case, diluted, or of full strength. When used, as I have used it often, of full strength, it causes only an exfoliation of the epidermis, no ulceration or destruction of any great amount of tissue in any case, and no pain or annoyance to the patient, the relief of the itching or presence of the distressing malady, producing so much satisfaction. It may be remarked that in sulphurous acid we have an agent as potent, but how long can we keep sulphurous acid as such? How often can we get sulphurous acid when prescribed? Were we at the door of a "Squibbs" with each patient, and had we the laboratory and conveniences of the expert and proficient chemist, and were qualified to make our own acid as we needed it, then probably we would need no addition to our therapeutics in the management of such skin diseases as these we are considering. In almost every case where I have resorted to the phenol prescription above, the patient has been a sufferer for many years, and has gone from one to the other of the profession.—*North Carolina Medical Journal, Dec., 1879.*

